# Supplementary material for: Region-Specific Homeostatic Identity of Astrocytes Is Essential for Defining Their Response to Pathological Insults
Source: Cells. 2023 Aug 30;12(17):2172. doi: 10.3390/cells12172172 (PMC10486627; doi:10.3390/cells12172172)
Supplement: Supplementary file 1 [file cells-12-02172-s001.zip › Tablee S1 - Key Resources.pdf]

Table S1 Key resources

REAGENTS	SOURCE	IDENTIFIER
**Antibodies**		
Anti-Iba1, rabbit polyclonal	FUJIFILM Wako Pure Chemical Corporation	#013-27691
Anti-GFAP, chicken polyclonal	Millipore Sigma	#AB5541
Goat anti-Rabbit, Alexa Fluor 546	Thermo Fisher Scientific	#A-11035
Goat anti-Chicken, Alexa Fluor 488	Thermo Fisher Scientific	#A-11039
**Chemicals**		
DAPI	Thermo Fisher Scientific	#62248
PBS, pH7.4	Thermo Fisher Scientific	#AM9624
10% buffered formalin	Millipore Sigma	#HT501128
Trizol	Thermo Fisher Scientific	#15596026
Chloroform	Millipore Sigma	#C2432
Ethanol (for RNA extraction)	Millipore Sigma	#E7023
Formic acid	Millipore Sigma	#F0507
Citrate Buffer, pH 6.0, Antigen Retriever	Millipore Sigma	#C9999
**Critical commercial assays**		
Aurum Total RNA Mini Kit	Bio-Rad	#7326820
Custom nCounter Mouse Astrocyte Panel	NanoString Technologies	UM_Astrocyte_1_C6977
**Experimental models**		
Mouse adapted prion strain SSLOW	Makarava et al., J Clin Invest., 2020	N/A
5XFAD (B6 SJLF1/J)	The Jackson Lab	JAX MMRRC Stock# 034840
**Software and algorithms**		
nSolver Analysis Software 4.0	NanoString Technologies	https://www.nanostring.com/products/analysis-solutions/ncounter-analysis-solutions/
nCounter Advanced Analysis (v. 2.0.115)	NanoString Technologies	https://www.nanostring.com/products/analysis-solutions/ncounter-advanced-analysis-software/
GraphPad Prism 9.2.0	GraphPad Software Inc	https://www.graphpad.com/scientific-software/prism/
Fiji ImageJ 1.53c	NIH	https://imagej.net/Fiji
