# Supplementary figures and images for: Region-Specific Homeostatic Identity of Astrocytes Is Essential for Defining Their Response to Pathological Insults
Source: Cells. 2023 Aug 30;12(17):2172. doi: 10.3390/cells12172172 (PMC10486627; doi:10.3390/cells12172172)

Figure S1

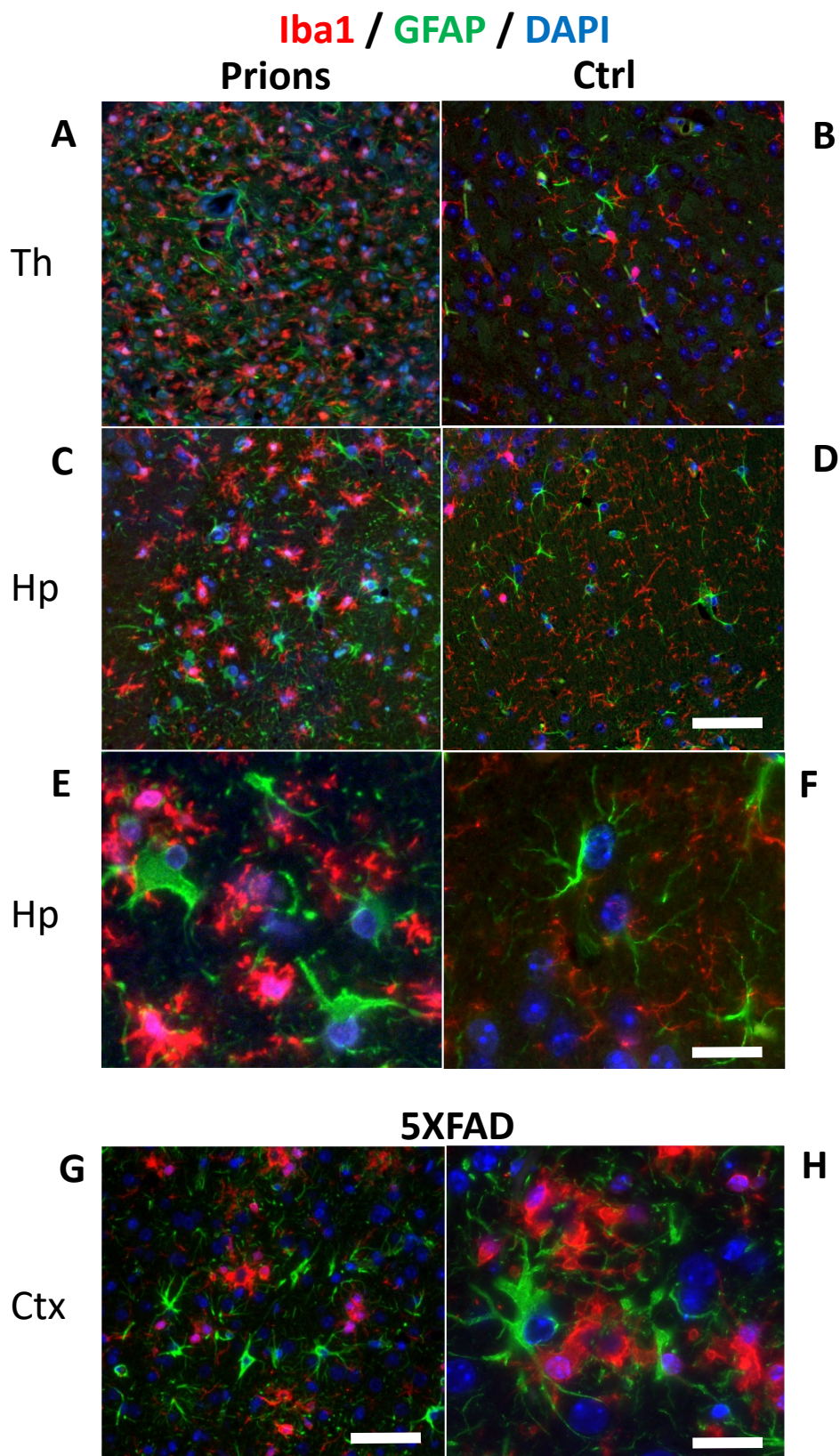

Figure S2

A

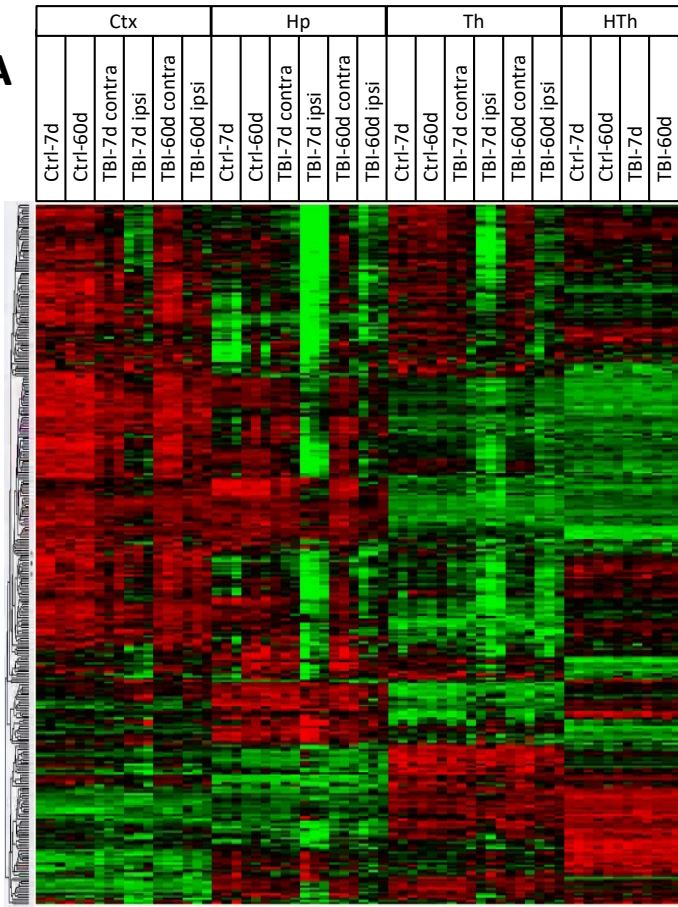

B

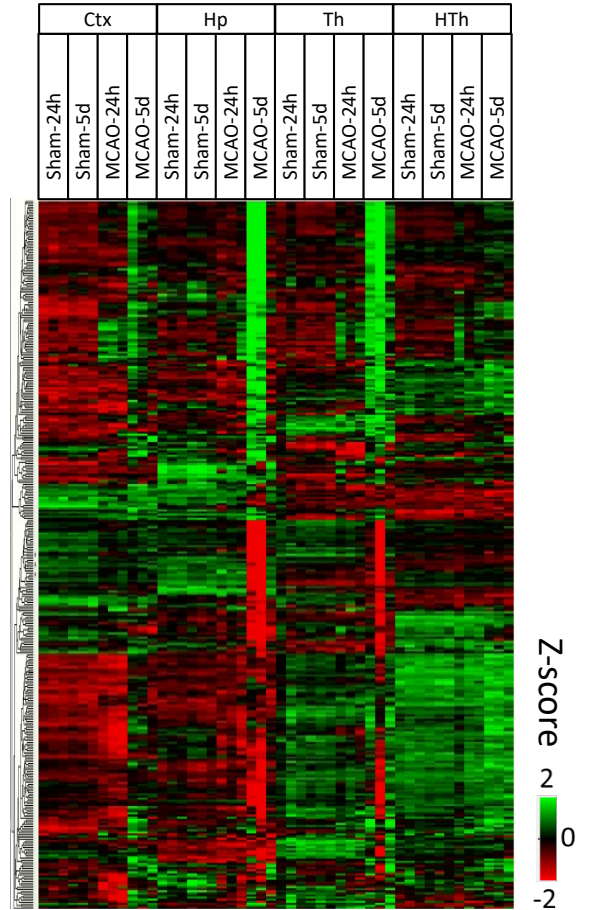

C

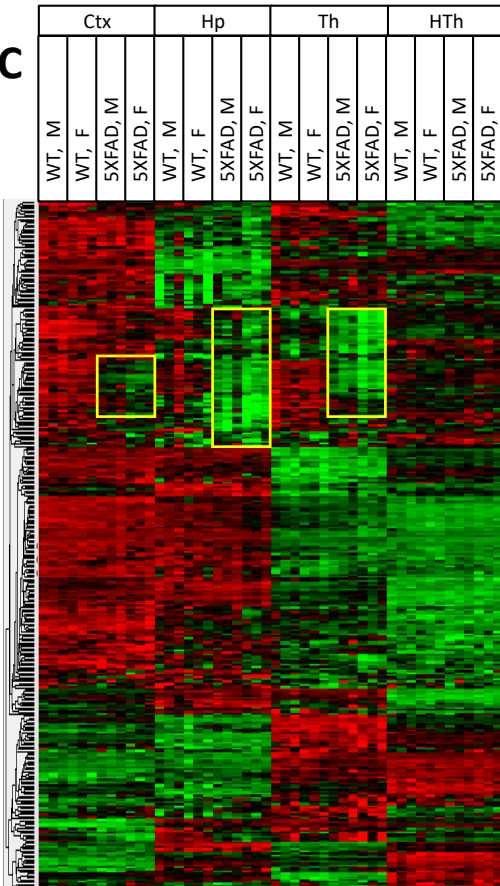

D

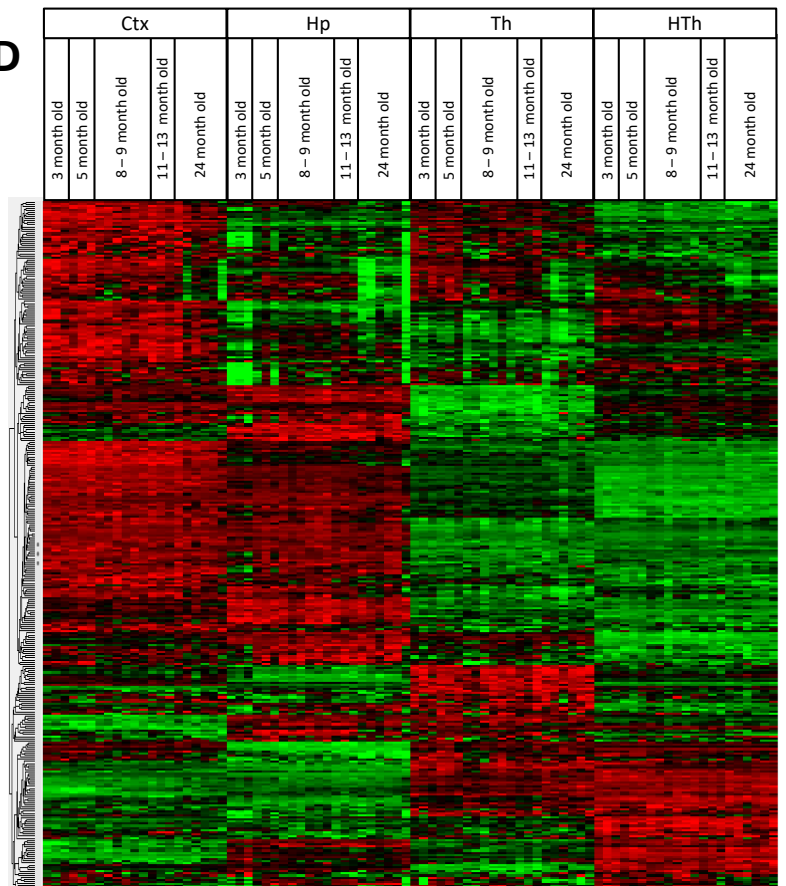

Figure S3

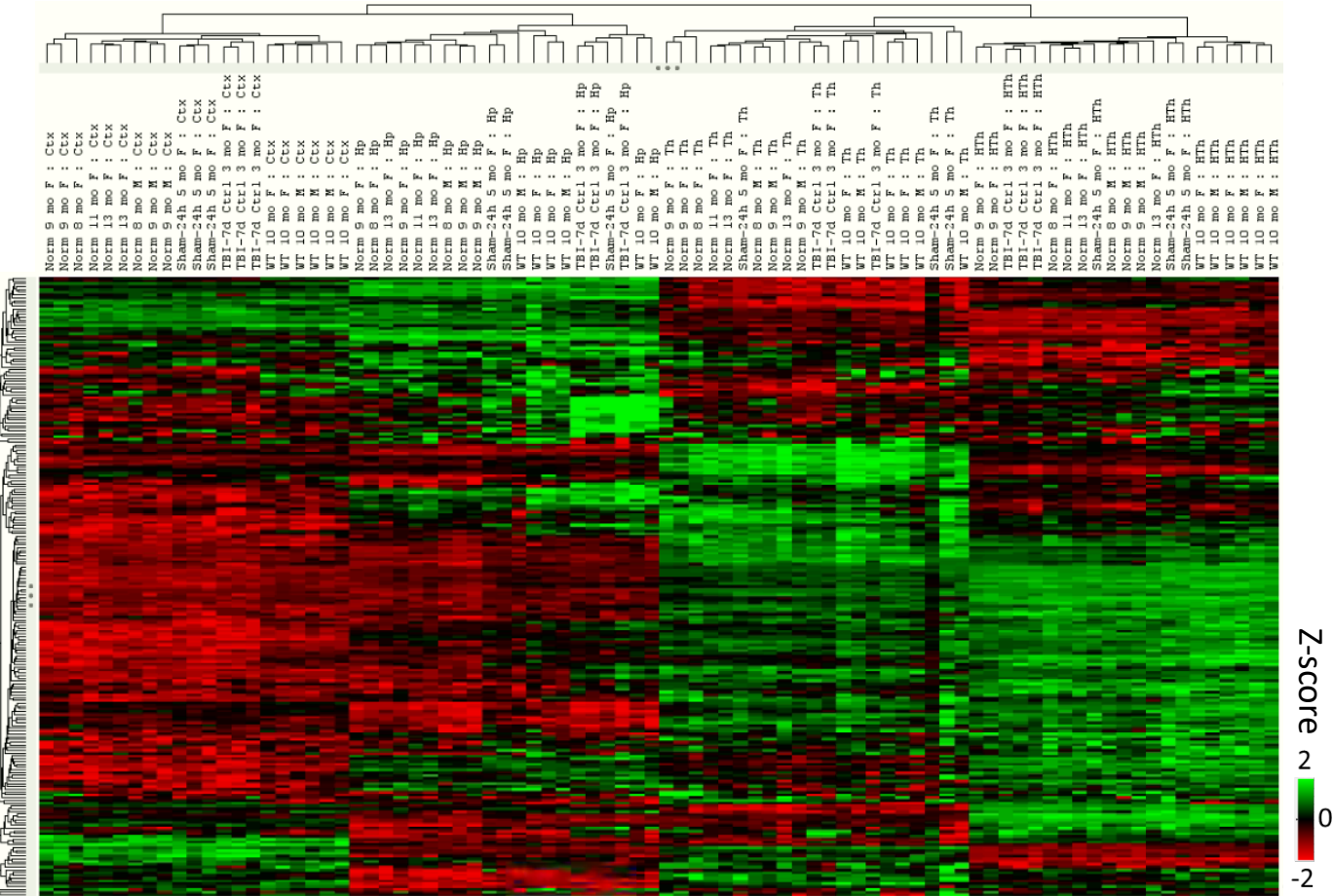

Figure S4

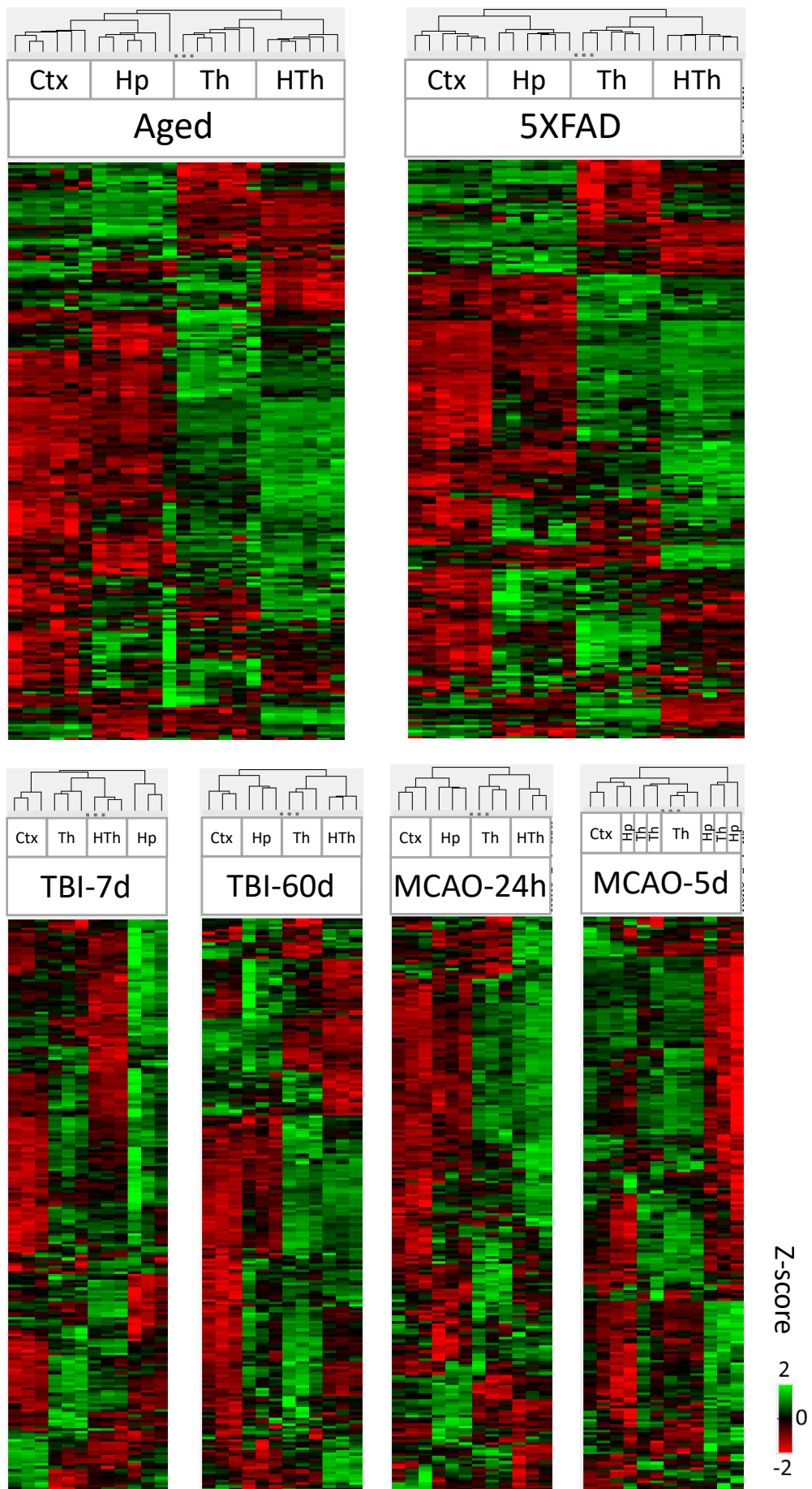

Figure S5

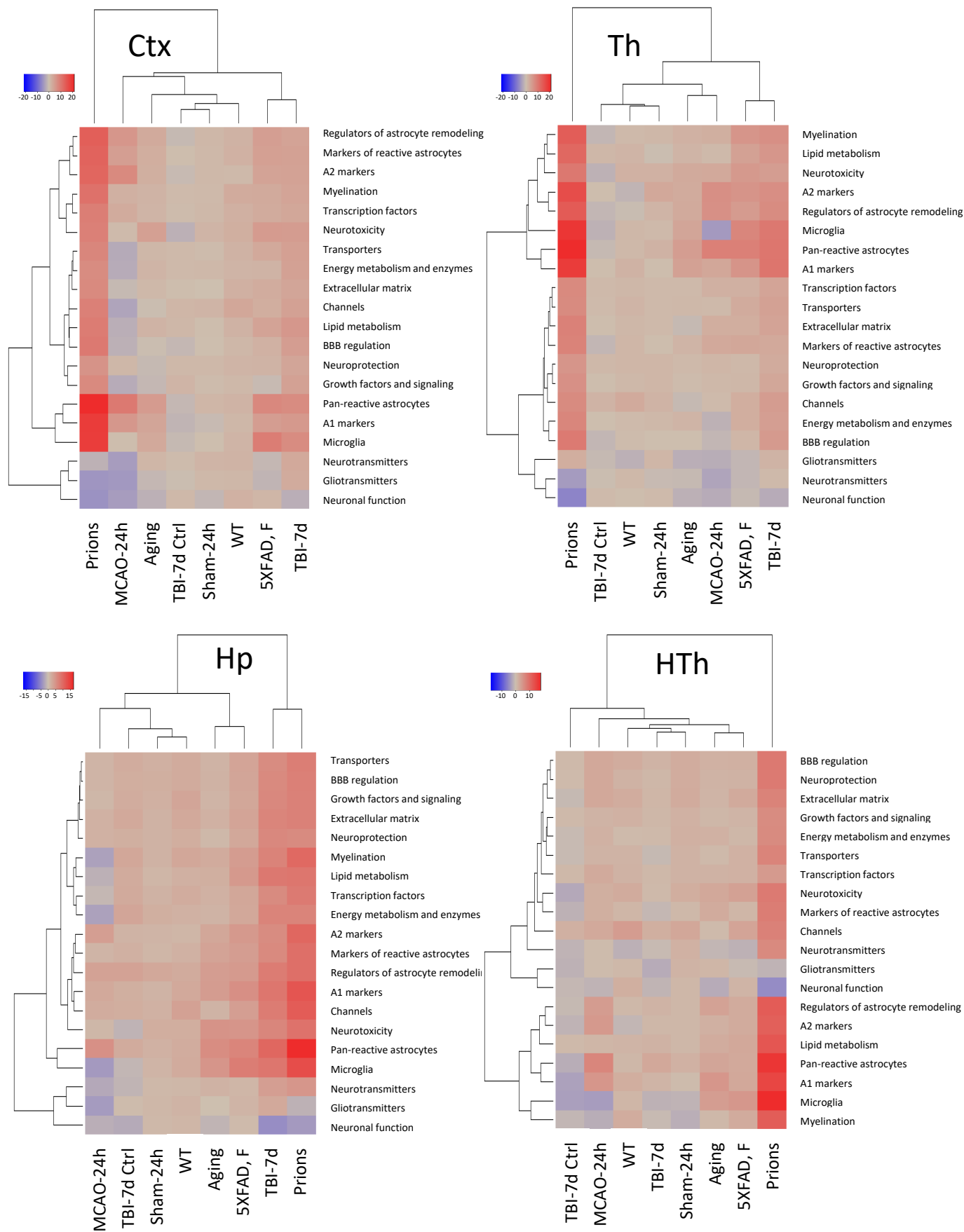

Figure S6

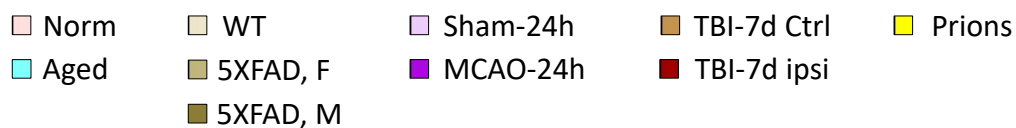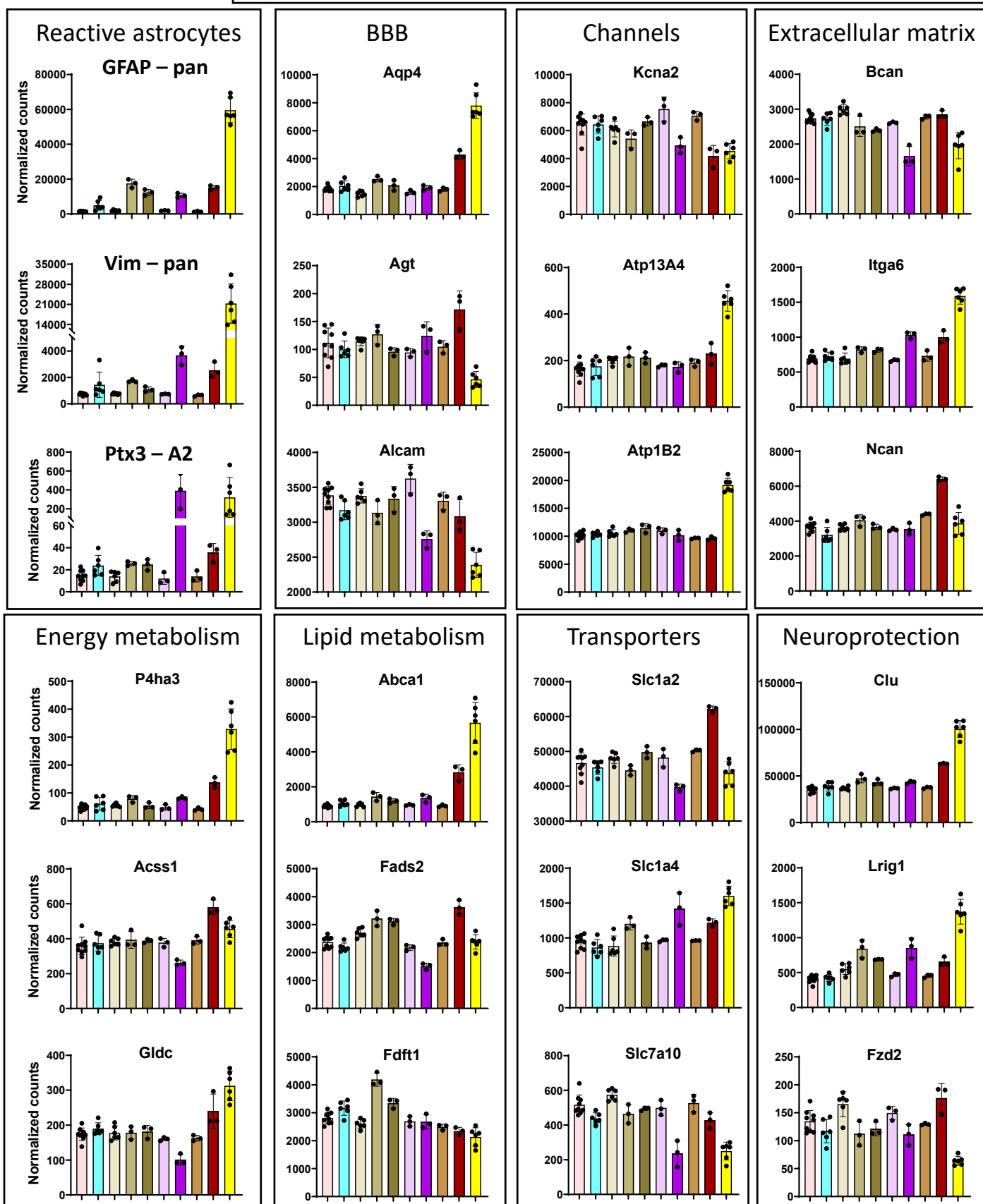

Supplement: Supplementary file 1 [file cells-12-02172-s001.zip › Figures-S1-S6.pdf]
